# The Strengths Use Scale: Psychometric Properties, Longitudinal Invariance and Criterion Validity

**DOI:** 10.3389/fpsyg.2021.676153

**Published:** 2021-06-15

**Authors:** Llewellyn E. van Zyl, Diane Arijs, Matthew L. Cole, Aldona Gliíska-Newes, Lara C. Roll, Sebastiaan Rothmann, Rebecca Shankland, Jacqueline M. Stavros, Nicolas B. Verger

**Affiliations:** ^1^Department of Industrial Engineering & Innovation Sciences, Human Performance Management, University of Eindhoven, Eindhoven, Netherlands; ^2^Optentia Research Focus Area, North-West University Vaal Triangle Campus (VTC), Vanderbijlpark, South Africa; ^3^Department of Human Resource Management, University of Twente, Enschede, Netherlands; ^4^Department of Social Psychology, Institut für Psychologie, Goethe University, Frankfurt, Germany; ^5^Department of Work and Organisation Studies, KU Leuven, Leuven, Belgium; ^6^College of Business and Information Technology, Lawrence Technological University, Southfield, MI, United States; ^7^Faculty of Economic Sciences and Management, Nicolaus Copernicus University, Torun, Poland; ^8^DIPHE, Department of Psychology, Education and Vulnerabilities, Université Lumière Lyon 2, Lyon, France; ^9^Department of Psychology, School of Life and Health Sciences, Glasgow Caledonian University, Glasgow, United Kingdom

**Keywords:** Strengths Use Scale, strengths assessment, psychometric properties, longitudinal invariance, positive psychological assessment, psychological strengths

## Abstract

Strengths use is an essential personal resource to consider when designing higher-educational programs and interventions. Strengths use is associated with positive outcomes for both the student (e.g., study engagement) and the university (e.g., academic throughput/performance). The Strengths Use Scale (SUS) has become a popular psychometric instrument to measure strengths use in educational settings, yet its use has been subjected to limited psychometric scrutiny outside of the U.S. Further, its longitudinal stability has not yet been established. Given the wide use of this instrument, the goals of this study were to investigate (a) longitudinal factorial validity and the internal consistency of the scale, (b) its equivalence over time, and (c) criterion validity through its relationship with study engagement over time. Data were gathered at two-time points, 3 months apart, from a sample of students in the Netherlands (*n* = 360). Longitudinal confirmatory factor analyses showed support for a two-factor model for overall strengths use, comprised of *Affinity for Strengths* and *Strengths Use Behaviors*. The SUS demonstrated high levels of internal consistency at both the lower- and upper bound limits at both time points. Further, strict longitudinal measurement invariance was established, which confirmed the instrument's temporal stability. Finally, criterion validity was established through relating strengths use to study engagement at different time stamps. These findings support the use of the SUS in practice to measure strengths use and to track the effectiveness of strengths use interventions within the higher education sector.

## Introduction

University students are three times more likely to develop psychopathological complaints and common mental health problems than the general population (Blanco et al., 2008; Seligman, 2012). This stems from severe psychological distress experienced as a result of an imbalance between their study demands (e.g., workload/time pressure), their study resources (e.g., lecturer support), and personal resources (e.g., strengths use; Lesener et al., 2020). The problem is exacerbated by intensive educational programmes, poor social relationships with peers (Houghton et al., 2018; Basson and Rothmann, 2019), drastic life changes, elevated levels of social comparison, peer pressure, and an imbalance between their studies and home life (Bergin and Pakenham, 2015). This, in turn, negatively affects students' motivation, study engagement, learning potential, academic performance, and overall academic throughput (Ebert et al., 2018). Therefore, it is not surprising that universities are implementing interventions to help students either (a) find a balance between their study demands/resources or (b) develop the internal personal resources needed to offset university life's impact on their well-being and academic performance (Seligman, 2012).

An essential personal resource targeted by these interventions relates to identifying and using personal strengths during one's studies. Strengths refer to the inherent psychological traits that students are naturally good at, leading to optimal functioning or performance in desired outcomes (Govindji and Linley, 2007). These are naturally occurring capacities that are universally valued by society (Huber et al., 2017). When students can live out their strengths during their studies, it could lead to positive outcomes for the self and others. Research shows that strengths are associated with positive self-esteem, goal achievement, pro-social behaviors, happiness, and well-being (Littman-Ovadia et al., 2017). Further, when students can live out their strengths at university, it also reduces reported levels of stress, depression, and anxiety (Schutte and Malouff, 2018). When students use their strengths during their studies, they are also more likely to perform academically and less likely to fall out of or change academic programmes (Seligman, 2012).

However, despite these positive associations, intervention studies centered around strengths-based development have shown mixed results (White, 2016; Roll et al., 2019; White et al., 2019). Although some strengths-based interventions have led to mental health and well-being changes, others did not (Quinlan et al., 2012; White et al., 2019). Van Zyl et al. (2019) argued that this is primarily because of poor intervention design and -measurement, where the focus is on measuring outcomes rather than on the underlying mechanisms being targeted by the intervention. In other words, strengths interventions aim to develop strengths *use*; however, what is ultimately measured is strengths possession or strengths knowledge. In fact, several studies have shown that only knowing what one's strengths are (strengths knowledge) is not enough to facilitate sustainable changes in positive individual outcomes (Seligman et al., 2005; Wood et al., 2011; Seligman, 2012; Proyer et al., 2015a,b; Miglianico et al., 2020). Only when one can actively apply one's strengths (i.e., strengths use) would it lead to happier and healthier lives (Govindji and Linley, 2007). Therefore, strengths use has become a central tenet in recent strengths-based intervention studies.

To measure such, Govindji and Linley (2007) developed the Strengths Use Scale (SUS), a 14 item self-report scale that aims to measure active strengths use. The instrument aims to measure both opportunities to use strengths (affinity for strengths use), as well as individual strengths, use behaviors (strengths use behaviors) (Van Woerkom et al., 2016a). The SUS is the most widely used instrument to assess general strengths use and has been translated into German (Huber et al., 2017), French (Forest et al., 2012), Hebrew (Littman-Ovadia et al., 2017), Finish (Vuorinen et al., 2020), Chinese (Bu and Duan, 2020), and even adapted to work settings (Dubreuil et al., 2014). Despite its wide use, only four studies have actively attempted to investigate its validity and reliability: Govindji and Linley (2007) and Wood et al. (2011) in the US, Huber et al. (2017) in Germany, and Duan et al. (2018) in China. Although all four studies have shown that SUS was a reliable and valid tool, those outside of the U.S. required several modifications (e.g., correlating error terms or item parceling) to ensure data-model fit. This trend is also prevalent in several empirical studies where the SUS was used (e.g., Mahomed, 2019; Mahomed and Rothmann, 2020; Vuorinen et al., 2020). Any form of statistical modification of a psychometric instrument fundamentally changes the content of what is being measured, thus limiting comparisons between studies (Price, 2017). As such, a thorough investigation as to the psychometric properties of the SUS is needed.

Therefore, the purpose of this study was to investigate the psychometric properties, longitudinal invariance, and criterion validity of the SUS within a student population. Specifically, it aimed to determine the (a) longitudinal factorial validity and the internal consistency of the instrument (b) its temporal equivalence, and (c) its relationship with strengths use and study engagement over time.

## Literature Review

### Conceptualization and Measurement of Strengths Use

Positive psychology is rooted in the tenet that individuals have inherent psychological strengths, which are activated to manage hardships and promote optimal human functioning (Peterson and Seligman, 2004). Strengths develop out of adversity and are *essential* to one's definition of self, are *effortless* in their enactment and *energizing* when activated (Matsuguma and Niemiec, 2020). Therefore, psychological strengths can be seen as positive, trait-like capacities that define good character and highlight “what is right” about an individual (Richter et al., 2020). These ideas are in line with Linley and Harrington's (2006, p. 86) definition of strengths as “natural capacities for behaving, thinking or feeling in a way that allows optimal functioning and performance in the pursuit of valued outcomes.” These capacities are universally valued by society as they lead to positive outcomes and benefits for both the self (e.g., positive mental health) and others (e.g., positive community climate) (Huber et al., 2017).

Further, research suggests that strengths are also relatively stable over time (Snow, 2019), are valued across cultures and educational contexts (McGrath, 2015), buffer against the onset of psychopathology (Peterson et al., 2006), enhance mental health (Seligman, 2012; Proyer et al., 2015a), and lead to context-specific positive outcomes such as study engagement, and academic performance (Kwok and Fang, 2020). Further, despite being relatively stable over time, strengths remain malleable and can be developed through interventions to promote strengths awareness and active strengths use (Huber et al., 2017).

Govindji and Linley (2007) argued that merely possessing a strength is not an effective means to promote personal growth and development. Instead, individuals need to both become aware- and develop a deep understanding of their strengths (i.e., strengths awareness/knowledge) and exert conscious effort to apply such in different situations (Wood et al., 2011). Strengths *awareness/knowledge* refers to the ability to know the things one is naturally good at and understand what role strengths play in one's daily life (Wood et al., 2011). On the other hand, strengths *use* refers to the extent to which one is both driven to apply and opportunities to use one's strengths in different situations (Wood et al., 2011; Van Woerkom et al., 2016a). Govindji and Linley (2007) conceptualization of strengths use is built on the organismic value process (OVP). The OVP proposes that strengths are naturally occurring traits that develop from within, where individuals are inherently driven to actively use, develop, apply, and play to their strengths in daily life. Further, individuals yearn to live by their strengths and are unconsciously drawn to activities, hobbies, studies, or work aligned to their strengths (Wood et al., 2011). Therefore, individuals are naturally drawn to activities aligned to their strengths (i.e., strengths affinity) and exhibit active strengths use behaviors (Wood et al., 2011; Van Woerkom et al., 2016a).

Although strengths possession and awareness/knowledge are shown to be important within the educational environment, intervention studies have shown that its indeed the conscious use of strengths that leads to sustainable changes in mental health and well-being over time (Wood et al., 2011; Seligman, 2012; Van Zyl and Rothmann, 2019; Miglianico et al., 2020). Govindji and Linley (2007) found that active strengths use leads to higher levels of happiness, personal fulfillment, and subjective- and psychological well-being. In contrast, strengths possession/awareness were not independent predictors of happiness or well-being (Seligman et al., 2005; Govindji and Linley, 2007). Albeit strengths awareness/possession is a precursor to active strengths use (Seligman et al., 2005). Despite these findings, most academic research has focused on the awareness-, identification- or possession of strengths, rather than the actual use thereof (Wood et al., 2011; Huber et al., 2017).

This is further indicated by the vast array of propriety psychometric instruments used to identify or assess strengths (Richter et al., 2020). These include, but are not limited to, the Clifton Strengths-Finder (Rath, 2007), the VIA Signature Strengths Inventory for adults (Peterson and Seligman, 2004) and children (Ruch et al., 2014), the Signature Strengths Questionnaire-72 (Rashid et al., 2017), the Personal Strengths Inventory (Kienfie Liau et al., 2011), the Realise2 Strengths Finder (Linley et al., 2010), and the Employee Strengths At Work Scale (Bhatnagar, 2020). Each of these instruments aims to measure various forms of manifested strengths ranging from character strengths to inherent talents. In contrast, only two psychometric instruments are available that measures strengths use: the Strengths-Use and Deficit Correction Behavior Scale (SUDCO; Van Woerkom et al., 2016a) and the Strengths Use Scale (SUS: Govindji and Linley, 2007; Wood et al., 2011).

The SUDCO aims to measure (a) strengths use behaviors, (b) deficit correction behaviors and perceived organizational support for (c) strengths use, and (d) -deficit correction (Van Woerkom et al., 2016a). Although this instrument has shown to be a valid and reliable tool to measure strengths use, it was crafted to be used within organizational settings (Van Woerkom et al., 2016a). This implies that the SUDCO cannot measure strengths use in other contexts (e.g., educational settings) or assess general strengths use behaviors or opportunities. Given that the SUDCO also focuses on deficit correction, the tool is not in line with the tenets of positive psychology (i.e., moving away from a focus on “fixing what is wrong,” but rather focus on developing what already works well; Seligman and Csikszentmihalyi, 2014). Further, the instrument is also not widely used within the literature (with only 71 citations on Google Scholar at the time of writing, i.e., early December 2020).

In contrast, the SUS is currently the most popular psychometric tool to measure strengths use behaviors and -opportunities within the literature with over a 1,000 citations (Govindji and Linley, 2007; Wood et al., 2011, p. 499). This 14 item self-report instrument aims to measure the extent to which individuals are drawn to activities that are aligned to their strengths and the extent to which strengths are actively used in a general way (Wood et al., 2011). The SUS has been translated into German (Huber et al., 2017), French (Forest et al., 2012), Hebrew (Littman-Ovadia et al., 2017), Finish (Vuorinen et al., 2020), Chinese (Bu and Duan, 2020), and adapted to work settings (Dubreuil et al., 2014). The instrument's popularity may be attributable to the fact that it was the first instrument developed to measure strengths use and that it is more inclined with the purest, functional principles of positive psychology.

### Factorial Validity of the Strengths Use Scale

The Strengths Use Scale (SUS) was initially developed as a self-report measure to understand the extent to which individuals can apply their strengths in daily life (Govindji and Linley, 2007). The instrument was developed around the idea that “strengths are natural, they come from within, and we are urged to use them, develop them, and play to them by an inner, energizing desire. Further, when we use our strengths, we feel good about ourselves, we are better able to achieve things, and we are working toward fulfilling our potential” (Linley and Harrington, 2006, p. 41). From this conceptualization, strengths use has both an active application component (strengths use behaviors) and encompasses opportunities to apply strengths to achieve personal goals or to facilitate personal development (opportunities to apply; Van Woerkom et al., 2016a).

Based on this conceptualization, Govindji and Linley (2007) generated 19 initial items, rated on a 7-point agreement type Likert scale, to measure strengths use from this perspective. Participants were instructed that these questions “*ask you about your strengths, that is, the things that you are able to do well or do best*” (Govindji and Linley, 2007, p. 147). A sample of 214 university students from the U.S. was requested to complete the SUS (Govindji and Linley, 2007). Principal component analysis revealed that three components with eigenvalues >1 could be extracted. However, the screen-plot showed that only a single component with 14 items could meaningfully be extracted from the data. These 14 items declared 56.2% of the total variance in a single “Strengths use” factor, with item loadings ranging from 0.52 to 0.79 (Govindji and Linley, 2007). The one-factor model showed to be significantly related to self-esteem, subjective well-being, psychological well-being, and subjective vitality, which established its concurrent validity (Govindji and Linley, 2007). However, this study only employed an exploratory approach, drawing a small sample from a single context. Therefore, the factorial validity could not formally be established nor verified. Despite showing promises, the authors argued that further validation studies on the SUS were needed.

In response, Wood et al. (2011) argued for the validation of the SUS within a general adult population (*N* = 227). This was done to increase the generalizability of the SUS within the U.S. Wood et al. (2011) employed both traditional factor analyses and parallel analyses to determine the factorial structure of the SUS. The results showed that a single strengths use factor could be extracted from the data based on eigenvalues. Items loaded between 0.66 and 0.87 on the single factor and declared 70.25% of the total variance.

Outside of the U.S., the SUS showed slightly different results. In the German validation, Huber et al. (2017) attempted to validate a translated version of the SUS within a sample of native German speakers. The authors employed both a traditional Exploratory Factor Analysis (EFA)- as well as a Confirmatory Factor Analysis (CFA) approach (through Structural Equation Modeling; SEM) to validate the instrument. The EFA showed that a single-factorial model, explaining 58.4% variance, with factor loadings ranging between 0.58 and 0.86, could be extracted from the data. The first factor had an eigenvalue of 8.60, with the remaining values clearly below the point of intersection (0.855–0.172). However, three items did not load sufficiently on the single strengths use factor (with factor loadings ranging from 0.336 to 0.410). The CFA was then conducted to determine if the hypothesized structure of the German SUS sample fitted the data well. However, the initial model fit of the German version was not satisfactory. Several modifications to the overall model needed to be implemented to enhance both model fit and measurement quality. This indicates that there may be conceptual overlap in understanding some items and that the factorial structure of the 14-items SUS may need further investigation.

### Internal Consistency of the SUS

Another factor to consider when considering the SUS as a viable and reliable tool to measure strengths use is its level of internal consistency or “reliability.” Reliability refers to the consistency and stability of an instrument to produce stable results (Wong and Wong, 2020). The SUS has shown to be a reliable measure across cultures; however, the level of internal consistency seems to vary within and between samples. In the original two U.S. validation studies, the SUS produced Cronbach's alpha coefficients ranging from 0.95 (Govindji and Linley, 2007) to 0.97 (Wood et al., 2011). Outside of the U.S., the SUS has shown acceptable levels of internal consistency in Germany (α = 0.84: Huber et al., 2017), China (α = 0.94: Bu and Duan, 2020), Finland (α = 0.88: Vuorinen et al., 2020), and the U.K. (α = 0.90: McTiernan et al., 2020).

Further, the test-retest reliability of the SUS was tested through intra-class correlations spanning three-time points (3 and 6 months after the first measurement). The test statistic was significant and very high (*r*_icc_ = 0.85), indicating that the SUS scores remained sufficiently stable without any specific intervention. Conversely, after a positive psychology intervention, strengths use scores have been shown to increase (e.g., Dubreuil et al., 2016), indicating that the scale is sensitive to measure changes.

However, despite the criticisms around Cronbach's alpha, only one other study employed a more restrictive and robust metric for internal consistency. Mahomed and Rothmann (2020) found that the composite reliability (i.e., upper bound level of internal consistency) of the SUS was 0.92. No other study specifically attempted to determine the upper level of internal consistency of the SUS.

### Stability of the SUS Over Time: Longitudinal Measurement Invariance

The temporal stability of the SUS is another essential metric to consider. This can be assessed through longitudinal measurement invariance (LMI). LMI is concerned with testing the factorial equivalence or equality of a construct over time (rather than across groups; Wong and Wong, 2020). Specifically, LMI assesses if the SUS produces similar factorial structures (configural invariance), if items load similarly on their respective factors (metric invariance), if the SUS shows to have similar intercepts (scalar invariance), and if similar residual errors are produced over time (Wong and Wong, 2020). LMI is a desirable characteristic of a measurement instrument as it provides evidence that a construct can be both measured and interpreted the same across different time stamps; therefore making meaningful interpretations and comparisons of mean scores of strengths use over time possible (Cheung and Rensvold, 2002; Widaman et al., 2010). No study has attempted to assess the LMI of the SUS over time, and therefore no specific reference points for such can be established from the current literature.

However, both Peterson and Seligman (2004) and Govindji and Linley (2007) argued that strengths are considered trait-like factors that are relatively stable over time. Further, the extent to which one would apply or use one's strengths is also considered stable over time, unless individuals are exposed to- or engage in strengths-based developmental initiatives (Seligman, 2012; Huber et al., 2017). Therefore, it is expected that strengths-use, without intervention, should stay relatively stable over time.

### Criterion Validity: Strengths Use and Study Engagement

A final metric to consider when validating an instrument is criterion validity. Criterion validity can be measured through establishing relationships with theoretically closely related variables (concurrent validity), and through the ability to predict outcomes on these related variables over time (predictive validity; Van Zyl, 2014). An important criterion to consider associated with active strengths use is study engagement (Ouweneel et al., 2011; Seligman, 2012; Stander et al., 2015; Kwok and Fang, 2020). Study engagement is a persistent and pervasive positive, fulfilling, and study-related state of mind characterized by feelings of vigor, showing dedication to one's studies and being absorbed in one's study-related tasks (Schaufeli et al., 2002). Drawing from desire theory, Seligman (2012) argued that when students can live in accordance with their strengths (i.e., engage in learning activities congruent with their strengths), or if they engage in study-related activities that are aligned to their strengths, that they will experience more engagement in their studies. The broaden-and-build theory of positive emotions further postulates that strengths are essential personal resources individuals can activate to translate positive emotional experiences into study-related engagement (Fredrickson, 2001). Several studies have also specifically shown that higher levels of active strengths-use lead to increased study and work-related engagement (Ouweneel et al., 2011; Seligman, 2012; Stander et al., 2015; Kwok and Fang, 2020). As such, both concurrent validity and predictive validity could be established by associating SUS with study engagement at different points in time.

### The Current Study

Given the importance of strengths use, and the popularity of the SUS within the literature, it is imperative to ensure that it is a valid and reliable instrument. As such, the purpose of this study was to investigate the psychometric properties, longitudinal invariance, and criterion validity of the Strengths Use Scale (SUS) within a student population. Specifically, the aim was to determine the: (a) longitudinal factorial validity and the internal consistency of the instrument, (b) its equivalence over time, and (c) criterion validity through its relationship with study engagement over time.

## Research Methods

### Research Approach

A quantitative, electronic survey-based longitudinal design was employed to determine the psychometric properties, longitudinal invariance and criterion validity of the SUS. This design entailed the distribution of questionnaires at two-time points over 3 months.

### Participants and Sampling Strategy

An availability-based sampling strategy was employed to draw 360 respondents from a University in The Netherlands to participate in this study. Table 1 provides an overview of the demographic characteristics of the sample. Validity responses were established by implementing two attention check items. If participants failed to score on these items, they were excluded from the analysis. As presented in Table 1, the majority of the participants were Dutch (97.8%) males (73.9%) between the ages of 20 and 22 years (78.9%) with a Bachelor's Degree (60.8%).

**Table 1:** Demographic characteristics of participants (*n* = 360).

**Item**	**Category**	**Frequency (*f)***	**Percentage (%)**
Gender	Male	266	73.9
	Female	93	25.8
	Other	1	0.3
Age (years)	20–22 years	284	78.9
	23–25 years	67	18.6
	23–30 years	8	2.2
Nationality	Dutch	352	97.8
	Other	8	2.2
Level of education	Bachelor's degree	219	60.8
	Master's degree	141	39.2

### Research Procedure

The data obtained for this paper are drawn from two large-scale cross-cultural student well-being projects. The Dutch sample consisted of two different datasets: one contained only third-year students and the other only master students. Data collection occurred during 2019-2020. The first cohort of data was collected between February to May 2019 and the second from November 2019 to January 2020 (before the COVID-19 outbreak). The period between measurements was 3 months. Online surveys were distributed at Time 1 and repeated at Time 2. A unique code was assigned to individuals to match Time 1 and Time 2 responses. Links were sent out to participants to their institutional email via Qualtrics^TM^ (www.qualtrics.com). In each survey, the rights and responsibilities of the participants were discussed. Participants provided online written informed consent. They were informed that their anonymity would be guaranteed and that their data would be stored in password-secured systems. Participants were informed they could withdraw their participation in this study at any time, without any repercussion for them. The purpose of the study was explained alongside the risks and benefits of the study. Participants' questions were answered at any step of the study.

### Measuring Instruments

The study made use of the three psychometric instruments.

A *demographic questionnaire* was used to gather basic biographic and demographic information about the participants. It aimed to capture respondents' self-identified gender identity, current age, nationality, home language, and level of education.

The *Strengths Use Scale* (SUS)^1^ developed by Govindji and Linley (2007) to measure how students actively used their strengths. The 14-item self-report questionnaire measured strengths use on an agreement-type Likert scale ranging from 1 (Strongly disagree) to 7 (Strongly agree) with items such as “I achieve what I want by using my strengths” and “Most of my time is spent doing things that I am good at doing.” The SUS showed acceptable levels of internal consistency at the lower bound limit with a Cronbach's alphas of 0.95 (Govindji and Linley, 2007).

The *Utrecht Work Engagement Scale* for students (UWES-9S) developed by Schaufeli et al. (2006) was used to measure study engagement. The 9-item questionnaire is rated on a six-point agreement type Likert scale ranging from 1 (Never) to 7 (Always). It measures the three components of study engagement with three items each. Example items are “When I am doing my work as a student, I feel bursting with energy” (vigor), “I am proud of my studies” (dedication), and “I get carried away when I am studying” (absorption). The UWES-9S has shown to be a valid and reliable measure in various contexts, with Cronbach Alpha's ranging from 0.72 to 0.93 (Schaufeli et al., 2006; Cadime et al., 2016).

### Statistical Analyses

Data were analyzed using SPSS v26 (IBM SPSS, 2019) and Mplus v 8.4 (Muthén and Muthén, 2020). A six-phased longitudinal factor analytical strategy through structural equation modeling was employed to investigate the psychometric properties, temporal stability, and concurrent/predictive validity of the SUS over time.

*First*, to explore the factorial structure of the SUS, an exploratory factor analytical (EFA) strategy was employed on the baseline data. To determine factorability, the Kaiser-Meyer-Olkin (KMO) measure and Bartlett's sphericity test was used. A KMO value larger than 0.60 and a statistically significant chi-square value on Barlett's test of sphericity would indicate that the data were factorable (Kaiser and Rice, 1974). Thereafter, an EFA was conducted through the structural equation modeling approach with the maximum likelihood estimation method and a Geomin (Oblique) rotation. Competing EFA factorial models were specified to be extracted based on Eigenvalues larger than 1 (Muthén and Muthén, 2020). Model fit statistics (c.f. Table 2) were used to establish data-model fit and to compare the competing EFA models. Further, items were required to load statistically significantly (Factor loading >0.40; *p* < 0.01) on their respective extracted factors and needed to declare at least 50% of the overall variance.

**Table 2:** Model fit statistics.

**Fit indices**	**Cut-off criterion**	**Sensitive to *N***	**Penalty for model complexity**
**Absolute fit indices**			
Chi-square (χ^2^)	Lowest comparative value between measurement models Significant (*p* > 0.01)	Yes	Yes
χ^2^/*df*	<3 = Excellent and <5 = Acceptable	No	No
**Approximate fit indices**			
Root-Means-Square Error of Approximation (RMSEA)	<0.08 but >0.01 90% CI Range doesn't include zero	Yes	Yes
Standardized Root Mean Square Residual (SRMR)	<0.08 but > 0.01	Yes	No
**Incremental fit indices**			
Comparative Fit Index (CFI)	>0.90 but <0.99	No	Yes
Tucker-Lewis Index (TLI)	>0.90 but <0.99	No	Yes
Akaike Information Criterion (AIC)	Lowest value in comparative measurement models	No	No
Bayes Information Criterion (BIC)	Lowest value in comparative measurement models	No	No

*Adapted from Kline (2011) and Wong and Wong (2020)*.

*Second*, a competing confirmatory factor analytical (CFA) measurement modeling strategy with the maximum likelihood estimation method (ML) was employed. As a baseline measure, three competing measurement models were specified and sequentially compared for each of the two-time points, separately. This approach verifies the best factorial structure and measurement quality of the instrument at each time point before evaluating temporal stability (Feldt et al., 2000). These separate and competing models were specified according to the traditional independent cluster model confirmatory factor analytical conventions where items were estimated to load onto their a priori theoretical factors and cross-loadings were constrained to zero (Wong and Wong, 2020)^2^.

To determine the best fitting measurement model at each time point and to mitigate the criticism of Hu and Bentler (1999) method of establishing model fit by *solely* looking at series of “cut-off points” and “standardized values” of fit indices, a sequential process of evaluation was implemented. As an initial step, the Hu and Bentler (1999) model fit criteria (c.f. Table 2) was used to determine data-model fit and to discriminate between measurement models for each time point. Thereafter, measurement quality was assessed through inspecting the standardized item loadings (λ > 0.40; *p* < 0.01), standard errors, item uniqueness (range between 0.1 and 0.9; *p* < 0.01), and the presence of multiple cross-loadings to further discriminate between models (Asparouhov and Muthén, 2009; Kline, 2011). Only models that showed both excellent fit *and*-measurement quality (with no items significantly loading on multiple factors) were retained for further analyses (McNeish and Hancock, 2018; McNeish et al., 2018; Shi et al., 2019).

*Third*, a longitudinal CFA (L-CFA) strategy was used to determine the temporal stability of the SUS's factorial structure. Here, the three measurement models from Time 1 were regressed on their corresponding counterparts in Time 2 (Von Eye, 1990). Again, these competing longitudinal measurement models were assessed for model fit/measurement quality and then systematically compared based on the same criteria as in the previous phase. As a first step in establishing temporal stability of the factorial models, two criteria needed to be met: (a) the regressive path between the factorial models of Time 1 and Time 2 were required to be large (Standardized β > 0.50) and statistically significant (*p* < 0.01) and (b) factorial models at Time 1 needed to declare at least 50% of the variance in its corresponding counterpart at Time 2 (Von Eye, 1990). The model that fit all the criteria was then retained for a more detailed item level inspection and further analyses.

*Fourth*, based on the best fitting L-CFA model, *item-level descriptive statistics, standardized factor loadings*, and *internal consistency* were investigated. Item related descriptive statistics were computed to provide a descriptive overview of each item in terms of means and standard deviations, inspect the corrected item-total correlations (CITC), and determine absolute normality (Skewness and Kurtosis). Based on Kim (2013) suggestion, absolute values for Skewness (<2) and Kurtosis (<2) were used as indicators of normality as our sample size was smaller than 500. The CITC represents the relationship of each item to the overall SUS, where correlations of less than *r* = 0.30 indicate that an item may not represent the overall factor (Zijlmans et al., 2019). Subsequently, point-estimate reliability (upper-bound; ρ > 0.80; Raykov, 2009), Mc Donald's Omega (ω > 0.80; Hayes and Coutts, 2020) and Cronbach's alpha (lower-bound; α > 0.70; Nunnally and Bernstein, 1994) for the best fitting model were computed to determine the internal consistency of the SUS and its subscales. Further, the average variance extracted (AVE) acts as an indicator for the average reliability of each individual indicator (item) in a scale, where a value over 50% is acceptable (Kline, 2011).

*Fifth*, second-order *longitudinal measurement invariance* (LMI) was implemented to determine whether the SUS is measured similarly at Time 1 and Time 2. LMI was assessed through applying increasingly restrictive equality constraints on the best fitting (second-order) L-CFA through estimating:

1. configural invariance (similar factor structures at baseline).
2. metric invariance for the first-order factorial model (similar factor loadings over time).
3. metric invariance for the second-order factorial model.
4. scalar invariance for the first-order factorial model (similar intercepts over time).
5. scalar invariance for the second-order factorial model.
6. strict invariance for the overall model (similar residual errors over time).

Invariance was established by comparing these ever-restrictive models on predefined criteria (Chen, 2007). A chi-square difference test was first computed but not used due to its sensitivity to minor parameter changes in small samples and model complexity (Cheung and Rensvold, 2002; Chen, 2007; Widaman et al., 2010). Instead, changes in RMSEA (Δ <0.015), SRMR (Δ <0.015), CFI (<0.01), TLI (<0.01), and chi-square/df (<1) indicated invariance (Cheung and Rensvold, 2002; Widaman et al., 2010). For comparisons, the least restrictive model was compared to the increasingly constrained models in each sequential step of the estimation process. If invariance was established, latent mean differences between the time points could be computed. Here, the Time 1 mean score was constrained to zero and used as the reference group. Time 2 mean score was freely estimated. Should Time 2 latent mean score differs significantly from zero, it would indicate a significant difference between timestamps (Wickrama et al., 2016; Wong and Wong, 2020).

Finally, to establish *concurrent and predictive validity*, separate structural models were estimated with the best fitting L-CFA model as an exogenous factor and study engagement as the endogenous factor. For concurrent validity, Strengths Use at Time 1 was regressed on Study Engagement Time 1 and Strengths Use Time 2 regressed on Study Engagement Time 2. To establish predictive validity, Strengths Use Time 1 was regressed on Study Engagement Time 2. A significance level of p <0.01 (99% confidence interval) for each regressive path.

## Results

The results of the exploratory factor analyses, baseline competing measurement models, longitudinal factor analyses, item-level descriptive (and internal consistency), longitudinal measurement invariance, and concurrent/predictive validity are reported separately in this section. The results are presented in a tabulated format with brief subsequent interpretations.

### Exploratory Factor Analysis

To explore the factorial structure of the SUS an EFA approach was employed on the baseline data. First, factorizability was established through the KMO measure and Bartlett's test for sphericity. The results showed that the KMO value was larger than 0.60 (KMO = 0.94) and produced a significant chi-square (*p* < 0.01). Meaningful factors could therefore be extracted, and we proceeded to estimate the EFA models.

As an initial measure, one to five factorial models were specified to be extracted. The results showed that two factors could be extracted with eigenvalues larger than 1. Further, only two models converged: A *single first order factorial model* ($\chi_{\left(360\right)}^{2}$ = 391.48; *df* = 77; χ^2^/*df* = 5.08; CFI = 0.89; TLI = 0.87; RMSEA = 0.11 [0.097,0.118]; SRMR = 0.05; AIC= 12588.99; BIC = 12751.73; Eigenvalue: 7.55; *R*^2^ = 53.94%) and a *two first order factorial model* ($\chi_{\left(360\right)}^{2}$ = 228.96; *df* = 64; χ^2^/*df* = 3.58; CFI = 0.94; TLI = 0.92; RMSEA = 0.08 [.073,0.097]; SRMR = 0.03; AIC = 12452.47; BIC = 12665.59; Eigenvalue Factor 1 = 7.55; *R*^2^= 53.94%; Eigenvalue Factor 2 = 1.05; R^2^= 7.48%). Only the two first-order factorial model fitted the data. This model showed significantly better fit than the single first-order factorial model. The item loadings and declared variance for this model are presented in Table 3^3^. All items loaded larger than 0.40 onto their respective factors. The first factor was labeled *Affinity for Strengths* (“Affinity”) and the second factor as *Strengths Use Behaviors* (“Active Use”). The Geomin factorial correlation showed that Affinity and Active Use were strongly correlated (*r* = 0.73; *p* < 0.01).

**Table 3:** EFA: Geomin rotated factor loadings and declared variance.

	**Item**	*****λ**_1_***	*****λ**_2_***	**R^**2**^ (%)**
**Affinity**				7.48
STU_1	I am regularly able to do what I do best	0.80	−0.01	
STU_2	I pursue goals and activities that are aligned to my strengths	0.83	−0.01	
STU_3	[Redacted]	0.48	0.21	
STU_4	[Redacted]	0.53	0.25	
STU_7	[Redacted]	0.58	0.07	
STU_12	Most of my time is spent doing the things that I am good at doing	0.41	0.26	
**Active use**				63.94
STU_5	I use my strengths everyday	0.19	0.59	
STU_6	I use my strengths to get what I want out of life	0.27	0.53	
STU_8	[Redacted]	0.21	0.54	
STU_9	[Redacted]	−0.09	0.81	
STU_10	I find it easy to use my strengths in the things I do	−0.02	0.81	
STU_11	[Redacted]	0.05	0.78	
STU_13	Using my strengths is something I am familiar with	0.13	0.65	
STU_14	[Redacted]	−0.00	0.81	

### Cross-Sectional Factorial Validity: Competing Measurement Models for Time 1 and Time 2

A competing measurement modeling strategy was employed to establish the factorial validity of the SUS on each of the “cross-sectional” data points. Here, observed items were used as indicators of latent factors. No items were removed and error terms were permitted to correlate.

The following models were estimated separately at both Time 1 and Time 2:

- **Model 1 & Model 4**: A one-factor first-order factorial model was estimated where all 14 items loaded directly on to a single factor called “Overall Strengths Use.”
- **Model 2 & Model 5**: Two correlated first-order factor models were estimated for a factor labeled “Strengths Affinity” (comprised of items 1, 2, 3, 4, 7, 12) and “Active Use” (comprised out of items 5, 6, 8, 9, 10, 11, 13, 14).
- **Model 3 & 6**: A second-order factorial model comprised out of the two first-order factors specified in the previous model was specified to directly load onto overall Strengths Use.

Table 4 presents the model fit indices for each of the estimated models. At ***Time 1***, the results showed that only Models 2 and 3 fitted the data ($\chi_{\left(360\right)}^{2}$ = 267.48; *df* = 76; χ^2^/*df* = 3.52; CFI = 0.93; TLI = 0.92; RMSEA = 0.08 [0.073,0.095]; SRMR = 0.04). Both models further fitted the data significantly better than Model 1 (Δχ^2^ = −124.00; Δ*df* = −1; χ^2^ /*df* = −1.56; ΔCFI = 0.04; ΔTLI = 0.05; ΔRMSEA = −0.03; ΔSRMR = −0.01; ΔAIC: −122.00; ΔBIC: −118.13).

**Table 4:** Cross-sectional confirmatory factor analyses: measurement model fit statistics for time 1 and time 2.

**Model**	**Description**	**χ^2^**	***df***	**χ^2^ / *df***	**CFI**	**TLI**	**RMSEA**		**SRMR**	**AIC**	**BIC**	**aBIC**	**Meets criteria**
**Time 1**													
Model 1	One-factor model	391.48	77	5.08	0.89	0.87	0.11	[0.097, 0.118]	0.05	12588.99	12751.74	12618.50	No
Model 2	Two-factor model	267.48	76	3.52	0.93	0.92	0.08	[0.073, 0.095]	0.04	12466.99	12633.61	12497.20	Yes
Model 3	Second-order factor model	267.48	76	3.52	0.93	0.92	0.08	[0.073, 0.095]	0.04	12466.99	12633.61	12497.20	Yes
**Time 2**													
Model 4	One-factor model	362.50	77	4.71	0.91	0.89	0.10	[0.092, 0.114]	0.04	11526.02	11688.17	11554.93	Partially
Model 5	Two-factor model	328.41	76	4.32	0.92	0.91	0.10	[0.087, 0.108]	0.04	11493.92	11659.94	11523.52	Yes
Model 6	Second-order factor model	328.41	76	4.32	0.92	0.91	0.10	[0.087, 0.108]	0.04	11493.92	11659.94	11523.52	Yes

*χ^2^, Chi-square; df, degrees of freedom; TLI, Tucker-Lewis Index; CFI, Comparative Fit Index; RMSEA, Root Mean Square Error of Approximation [90%CI]; SRMR, Standardised Root Mean Square Residual; AIC, Akaike Information Criterion; BIC, Bayes Information Criterion; aBIC, Adjusted Bayes Information Criterion*.

The result showed a similar pattern at ***Time 2***, only Models 5 and 6 fitted the data ($\chi_{\left(360\right)}^{2}$ = 328.40; *df* = 76; χ^2^/*df* = 4.32; CFI = 0.92; TLI = 0.91; RMSEA = 0.10 [0.087,0.108]; SRMR = 0.04). Both models fitted the data significantly better than Model 4 (Δχ^2^ = −34.10; Δ*df* = −1; χ^2^ /*df* = −0.56; ΔCFI = 0.03; ΔTLI = 0.02; ΔRMSEA = −0.00; ΔSRMR = 0.00; ΔAIC: −31.41; ΔBIC: −28.23).

In respect of measurement quality, all models at both Time 1 and Time 2 showed acceptable levels with standardized factor loadings (λ > 0.40; *p* < 0.01), standard errors, and item uniqueness (δ <0.10 but > 0.90; *p* < 0.01) meeting the classification criteria (Asparouhov and Muthén, 2009; Kline, 2011).

### Longitudinal Factor Analyses: Longitudinal Factorial Validity and Temporal Stability

The next step in the process was to determine the stability of the SUS over time using L-CFA. In each L-CFA model, the corresponding measurement model specified in Time 1 was regressed on Time 2. The following models were tested:

- **Model 7:** The single, first-order factor (with all 14 items loading directly on to such) of Time 1 was regressed on the single first-order factor of Time 2.
- **Model 8:** The two first-order factor models of “Strengths Affinity” (comprised of items 1, 2, 3, 4, 7, 12) and “Active Use” (comprised out of items 5, 6, 8, 9, 10, 11, 13, 14) at Time 1 was regressed onto their corresponding Strengths Affinity and Proactive Use factorial counterparts. Covariances between the factors at each time point was permitted.
- **Model 9:** The second-order factorial model of Time 1 was regressed on that of Time 2 was regressed. Both models comprised out of the two first-order factors specified in the previous model. Covariances between the factors at each time point was not permitted. Error terms on Item 14 and 11 were permitted to covary at Time 2.

The results summarized in Table 5 indicated that only Model 9, the second-order longitudinal factorial model, fitted the data ($\chi_{\left(360\right)}^{2}$ = 974.93; *df* = 344; χ^2^/*df* = 2.83; CFI = 0.90; TLI = 0.90; RMSEA = 0.07 [0.066,0.077]; SRMR = 0.04). Model 9 also fitted the data significantly better than Model 7 (Δχ^2^ = −224.31; Δ*df* = −5; χ^2^ /*df* = −0.60; ΔCFI = 0.03; ΔTLI = 0.04; ΔRMSEA = −0.01; ΔSRMR = 0.00; ΔAIC: −214.20; ΔBIC: −194.77) and Model 8 (Δχ^2^ = −53.58; Δ*df* = −2; χ^2^ /*df* = −0.14; ΔCFI = 0.00; ΔTLI = 0.01; ΔRMSEA = 0.00; ΔSRMR = 0.00; ΔAIC: −49.58; ΔBIC: −41.81). All longitudinal models showed acceptable levels of measurement quality with standardized factor loadings (λ > 0.40; *p* < 0.01), standard errors, and item uniqueness (δ > 0.10 but <0.9; *p* < 0.01) exceeding the specified thresholds (Asparouhov and Muthén, 2009; Kline, 2011).

**Table 5:** Longitudinal confirmatory factor analyses: measurement model fit statistics for time 1 and time 2.

**Model**	**Description**	**χ^2^**	***df***	**χ^2^ /*df***	**CFI**	**TLI**	**RMSEA**		**SRMR**	**AIC**	**BIC**	**aBIC**	**Meets criteria**
Model 7	One-factor models	1199.14	349	3.44	0.87	0.86	0.08	[0.077, 0.0.87]	0.05	23941.81	24272.13	24002.47	No
Model 8	Two-factor models	1028.51	346	2.97	0.90	0.89	0.07	[0.069, 0.0.79]	0.05	23777.19	24119.17	23839.98	Partially
Model 9	Second-order factor models	974.93	344	2.83	0.90	0.90	0.07	[0.066, 0.077]	0.05	23727.61	24077.36	23791.83	Yes

*χ^2^, Chi-square; df, degrees of freedom; TLI, Tucker-Lewis Index; CFI, Comparative Fit Index; RMSEA, Root Mean Square Error of Approximation [90%CI]; SRMR, Standardised Root Mean Square Residual; AIC, Akaike Information Criterion; BIC, Bayes Information Criterion; aBIC, Adjusted Bayes Information Criterion*.

Further, to assess the final two assumptions for L-CFA, the regressive paths and covariances, as well as the variance declared by factorial models of Time 1 in Time 2, were estimated and summarized in Table 6. Although all the factors at Time 1 statistically significantly predicted the factors in Time 2, the results showed that only Model 9 met both the significance and variance criteria. The second-order factorial Strengths Use factor at Time 1 statistically significantly predicted 51% of the variance of Strengths Use in Time 2 with a large effect (β = 0.71; S.E = 0.03; *p* < 0.01). Therefore, only Model 9 was retained for further analyses.

**Table 6:** Longitudinal confirmatory factor analyses: regressive paths and covariances between time 1 and time 2.

**Model**	**Relational path**			**Standardized**						**Meets** ** criteria**
				**β**	***r***	**S.E**	***t*-value**	***p***	**R^**2**^**	
Model 7	Strengths use time 1	→	Strengths Use Time 2	0.68	-	0.03	20.63	0.00	0.46	Partially
Model 8	Affinity time 1	→	Affinity Time 2	0.66	-	0.04	17.22	0.00	0.44	Partially
	Active use time 1	→	Active Use Time 2	0.63	-	0.04	16.71	0.00	0.40	
	Affinity time 1	←→	Active Use Time 1	-	0.88	0.02	46.61	0.00	-	
	Affinity time 2	←→	Active Use Time 2	-	0.95	0.02	38.77	0.00	-	
Model 9	Strengths use time 1	→	Strengths Use Time 2	0.71	-	0.03	20.84	0.00	0.51	Yes

*→Regression, ←→Covariance*.

### Longitudinal Factor Loadings, Item Level Descriptive and Internal Consistency

Next, item-level descriptive statistics (means, standard deviations, skewness, kurtosis, CICT), standardized factor loadings, the Average Value Explained (AVE) and the level of internal consistency was computed for the second-order longitudinal factor model (Model 9). Table 7 provided a summary for the results.

**Table 7:** Item level descriptive statistics, standardized factor loadings, average value explained, and internal consistency for the longitudinal model 9.

**Factor**	**Item**	**Time 1–Second-order strengths use model (model 9)**												**Time 2–Second-order strengths use model (model 9)**											
		**x̄**	**σ**	**Skewness**	**Kurtosis**	**CITC**	**λ**	**S.E**.	**δ**	**AVE**	**ρ**	**α**	**ω**	**x̄**	**σ**	**Skewness**	**Kurtosis**	**CITC**	**λ**	**S.E**.	**δ**	**AVE**	**ρ**	**α**	**ω**
Affinity										0.50	0.86	0.85	0.85									0.54	0.87	0.87	0.87
	STU_1	5.20	1.05	−0.98	1.82	0.67	0.77	0.03	0.41	-	-	-		5.13	1.11	−0.73	0.23	0.73	0.78	0.02	0.40	-	-	-	
	STU_2	5.24	1.02	−0.70	0.85	0.70	0.79	0.02	0.38	-	-	-		5.20	0.90	−0.55	0.19	0.72	0.79	0.02	0.39	-	-	-	
	STU_3	5.59	0.81	−0.65	0.90	0.61	0.67	0.03	0.56	-	-	-		5.44	0.99	−0.73	0.53	0.71	0.76	0.03	0.43	-	-	-	
	STU_4	5.38	0.93	−0.66	0.79	0.70	0.75	0.03	0.43	-	-	-		5.26	0.88	−0.63	0.45	0.74	0.78	0.02	0.39	-	-	-	
	STU_7	4.77	1.62	−0.43	−0.15	0.56	0.63	0.04	0.61	-	-	-		4.92	1.28	−0.63	0.51	0.62	0.65	0.03	0.57	-	-	-	
	STU_12	4.68	1.53	−0.36	−0.36	0.60	0.63	0.04	0.60	-	-	-		4.89	1.13	−0.53	0.36	0.64	0.65	0.03	0.58	-	-	-	
Active Use										0.58	0.92	0.92	0.92									0.58	0.92	0.92	0.92
	STU_5	4.94	1.35	−0.56	0.36	0.71	0.75	0.03	0.44	-	-	-		5.04	1.28	−0.51	0.19	0.75	0.78	0.02	0.40	-	-	-	
	STU_6	5.22	1.29	−0.69	0.71	0.73	0.76	0.03	0.42	-	-	-		5.31	1.18	−0.61	0.36	0.74	0.77	0.02	0.40	-	-	-	
	STU_8	5.05	1.23	−0.48	−0.04	0.68	0.72	0.03	0.49	-	-	-		5.14	1.10	−0.30	−0.03	0.74	0.77	0.02	0.40	-	-	-	
	STU_9	5.17	1.41	−0.82	0.87	0.66	0.72	0.03	0.49	-	-	-		5.31	1.19	−0.63	0.30	0.70	0.74	0.03	0.45	-	-	-	
	STU_10	5.10	1.41	−0.71	0.29	0.72	0.78	0.02	0.40	-	-	-		5.18	1.03	−0.42	0.13	0.74	0.78	0.02	0.40	-	-	-	
	STU_11	5.10	1.20	−0.65	0.65	0.75	0.81	0.02	0.35	-	-	-		5.11	1.22	−0.51	0.10	0.74	0.76	0.03	0.42	-	-	-	
	STU_13	5.22	1.19	−0.63	0.43	0.71	0.74	0.03	0.45	-	-	-		5.28	1.10	−0.58	0.37	0.72	0.75	0.03	0.44	-	-	-	
	STU_14	5.03	1.19	−0.45	0.04	0.74	0.80	0.02	0.35	-	-	-		5.18	1.03	−0.40	0.08	0.77	0.78	0.02	0.39	-	-	-	
Strength-Use										0.85	0.92	0.93	0.93									0.95	0.97	0.94	0.94
	Affinity	-	-	-	-	-	0.90	0.03	0.19	-	-	-		-	-	-	-	-	0.98	0.02	0.04	-	-	-	
	Active Use	-	-	-	-	-	0.94	0.03	0.12	-	-	-		-	-	-	-	-	0.97	0.02	0.06	-	-	-	

*x̄, Mean; σ, Standard deviation; CICT, Corrected item total correlation; λ, Standardized factor loadings; S.E., Standard Error; δ, Item Uniqueness; ρ, Composite Reliability; α, Cronbach's Alph; ω:McDonald's Omega*.

The results of the item level descriptive statistics show that all items were normally distributed (Skewness and Kurtosis < +2;−2: Kim, 2013), that each item was clearly associated with the overall factor being assessed (CITC *r* > 0.30: Zijlmans et al., 2019) and that each sub-factor and overall strengths-use scale showed to be reliable at both the upper- (ρ > 0.80; ω > 0.80) and lower- bound level of internal consistency (α > 0.70) at both time points.

All items on Affinity and Active Use loaded statistically significantly on their respective factors at both time points with standardized factor loadings ranging from 0.56 to 0.81 (*p* < 0.01). The AVE for Affinity was acceptable, with 0.50 reported at Time 1 and 0.54 at Time 2. Similarly, the AVE for Active Use at Time 1 (AVE = 0.58) and Time 2 (AVE = 0.58) exceeded the 0.50 threshold.

Further, both the first-order Affinity (Time 1: λ = 0.90, SE = 0.03 *p* < 0.01; Time 2 = λ = 0.98 SE = 0.02; *p* < 0.01) and Active Use factors (Time 1: λ = 0.94, SE = 0.03, *p* < 0.01; Time 2: λ = 0.97, SE = 0.02, *p* < 0.01) loaded statistically significantly onto the second-order Strengths Use factor. The second-order longitudinal factorial model therefore showed to have an excellent level of measurement quality and can therefore be subjected to more robust assessments of longitudinal stability over time.

### Longitudinal Measurement Invariance and Mean Comparisons

Next, longitudinal measurement invariance (LMI) was tested to determine the factorial equivalence of the SUS over time. The results, summarized in Table 8, showed that all invariance models fitted the data based on the criteria mentioned in Table 2 and that longitudinal measurement invariance of the SUS could be established between the different time points. No significant differences in terms of RMSEA (Δ <0.015), SRMR (Δ <0.015), CFI (<0.010), TLI (<0.010), and χ^2^*/df* (<1) between the configural, metric, scalar, and strict invariance models were found (Cheung and Rensvold, 2002; Widaman et al., 2010; Wong and Wong, 2020). Therefore, the SUS showed to be a consistent measure over time and that meaningful mean comparisons between Time 1 and Time 2 can be made.

**Table 8:** Longitudinal measurement invariance for the second-order strengths use model (model 9).

	**Model**	**χ^2^**	***df***	**χ^2^*/ df***	**CFI**	**TLI**	**RMSEA**		**SRMR**	**Model comparison**	***Δχ*^2^**	**Δ **χ^2^***/ df***	**ΔCFI**	**ΔTLI**	**ΔRMSEA**	**ΔSRMR**	**Meets criteria**
**M1**	Configural invariance	850.49	330	2.58	0.921	0.909	0.066	[0.061, 0.072]	0.043	-	-	-	-	-	-	-	Yes
**M2**	Metric invariance: first-order	868.17	342	2.54	0.919	0.911	0.065	[0.060, 0.071]	0.052	M2 vs. M1	17.68	−0.039	−0.002	0.002	0.001	0.009	Yes
**M3**	Metric invariance: second-order	879.19	343	2.56	0.918	0.909	0.066	[0.061, 0.071]	0.053	M3 vs. M2	11.02	0.025	−0.001	−0.002	0.001	0.001	Yes
**M4**	Scalar invariance: first-order	917.75	356	2.58	0.914	0.909	0.066	[0.061, 0.071]	0.057	M4 vs. M3	38.56	0.015	−0.004	0.000	0.000	0.004	Yes
**M5**	Scalar invariance: second-order	927.51	355	2.61	0.912	0.907	0.067	[0.062, 0.072]	0.057	M5 vs. M4	9.76	0.035	−0.002	−0.002	0.001	0.000	Yes
**M6**	Strict invariance	942.64	358	2.63	0.910	0.905	0.067	[0.062, 0.073]	0.060	M6 vs. M5	15.13	0.020	−0.002	−0.002	0.000	0.003	Yes

*χ^2^, Chi-square; df, degrees of freedom; TLI, Tucker-Lewis Index; CFI, Comparative Fit Index; RMSEA, Root Mean Square Error of Approximation [90%CI]; SRMR, Standardised Root Mean Square Residual*.

Further, to compare latent means on the first- and second-order factors of the SUS, all mean scores at Time 1 were constrained to zero within the strict invariance model. Affinity, Active Use, and Overall Strengths Use at Time 2 were then freely estimated. For the first-order factors, the results showed that Affinity (Δ x̄ = −0.7; SE = 0.04; *p* = 0.10) and Active Strengths Use (Δ x̄ = 0.7; SE = 0.05; *p* = 0.11) at Time 2 did not meaningfully differ from Time 1. Similarly, at a second-order factorial level, Overall Strengths Use at Time 2 (Δ x̄ = 0.0; SE = 0.04; *p* = 0.908) did also not meaningfully differ from Time 1.

### Concurrent and Predictive Validity

To establish concurrent and predictive validity, separate structural models were estimated with the second-order Strengths Use models specified as an exogenous factor and Study Engagement (as a second-order factor made up of three first-order factors: Vigor, Dedication, and Absorption) specified as endogenous factors. The results for both concurrent and predictive validity are summarized in Table 9.

**Table 9:** Concurrent and predictive validity of the second-order strengths use model on study engagement.

**Model**	**Regressive path**			**Standardized**					**Validity established**
				**β**	**S.E**	***t*-value**	***p***	**R^**2**^**	
Concurrent validity	Strengths use time 1	→	Study engagement time 1	0.49	0.05	9.58	0.00	0.24	Yes
	Strengths use time 2	→	Study engagement time 2	0.58	0.04	13.54	0.00	0.33	Yes
Predictive validity	Strengths use time 1	→	Study engagement time 2	0.47	0.05	9.18	0.00	0.22	Yes

*→Regression*.

For *concurrent validity*, Strengths Use at Time 1 was first was regressed on Study Engagement at Time 1. The model showed adequate fit ($\chi_{\left(360\right)}^{2}$ = 595.83; *df* = 225; χ^2^/*df* = 2.65; CFI = 0.92; TLI = 0.91; RMSEA = 0.06 [0.061,0.075]; SRMR = 0.06; AIC = 20707.47; BIC = 20994.22). Strengths Use at Time 1 was directly associated with Study Engagement at Time 1 (β = 0.49; S.E = 0.05; *p* < 0.01; *R*^2^ = 0.24). Similarly, Strengths Use at Time 2 was also directly associated with Study Engagement at Time 2 (β = 0.58; S.E = 0.04; *p* < 0.01; R^2^ = 0.33). This model also showed adequate fit ($\chi_{\left(360\right)}^{2}$ = 689.80; *df* = 225; χ^2^/*df* = 3.07; CFI = 0.91; TLI = 0.90; RMSEA = 0.08 [0.070,0.083]; SRMR = 0.06; AIC = 19403.58; BIC = 19689.28).

For *predictive validity*, Strengths Use at Time 1 was regressed on Study Engagement at Time 2. This model showed adequate fit ($\chi_{\left(360\right)}^{2}$ = 576.37; *df* = 225; χ^2^/*df* = 3.07; CFI = 0.93; TLI = 0.91; RMSEA = 0.07 [0.069,0.073]; SRMR = 0.06; AIC = 20423.66; BIC = 20711.24). Here, Strengths Use at Time 1 predicted 22% of the variance in Study Engagement at Time 2 (β = 0.47; S.E = 0.05; *p* < 0.01; *R*^2^: 0.24). Both concurrent and predictive validity of the SUS could therefore be established.

## Discussion

This study aimed to investigate the psychometric properties, longitudinal invariance, and criterion validity of the SUS within a Dutch student population. Longitudinal confirmatory factor analysis showed that a second-order factorial model, comprised of two first-order factors (*Affinity for Strengths* and *Strengths Use Behaviors*), fitted the data best. Further, this model showed support for strict longitudinal measurement invariance over 3 months with similar factorial structures, -factor loadings, item intercepts, and item uniqueness. Further, the SUS produced high levels of internal consistency at both the lower- and upper bound limits at both time stamps. Mean comparisons showed that neither overall strengths use, nor its two components, differed between Time 1 and Time 2. This confirmed the stability of the SUS over time. Finally, strengths use was related to study engagement at both time points. Strengths use at Time 1 also predicted study engagement at Time 2. Therefore, supporting the assumptions of criterion validity.

### The Psychometric Properties of the Strengths Use Scale

Longitudinal factor analyses showed that a second-order factorial model of overall strengths use, comprising two first-order factors called *Affinity for Strengths* and *Strengths Use Behaviors*, fitted the data. *Affinity for Strengths* comprised six items related to opportunities where individuals could live out or apply their strengths. These opportunities related to activities that individuals are drawn to and that are naturally aligned to their strengths (Wood et al., 2011; Van Woerkom et al., 2016a). Individuals seek out activities where they can both live out- and pursue goals aligned to their strengths. They further show a natural affinity for mastering new skills/hobbies where these strengths are required (Govindji and Linley, 2007).

On the other hand, active *Strengths Use Behaviors* was measured by eight items related to the behaviors' individuals exhibit when applying strengths in everyday life. These behaviors related to actions employed by individuals to actively develop and apply their strengths to achieve life goals. Here, individuals can actively deploy their strengths to get what they want out of life (Govindji and Linley, 2007).

This two-factorial permutation of the SUS contrasts with Govindji and Linley (2007) and Wood et al. (2011), who reported strengths use as a single, first-order factor. Although our findings contrast with these authors' empirical results, it is in line with the original theoretical tenet on which the instrument was built. Govindji and Linley (2007) argued that strengths use is a function of the organismic value process and the self-concordant goal theory (from which items of the SUS was generated). According to Joseph and Linley (2005), the organismic value process suggests that strengths are psychological traits that individuals are inherently driven to use, develop, and apply (i.e., behaviors). Further, individuals express an inherent desire to live by their strengths and are unconsciously attracted to and show an affinity for activities/hobbies, studies, or work that are aligned to their strengths (i.e., affinity) (Wood et al., 2011; Huber et al., 2017). Therefore, our results are more closely aligned to the original theoretical ideas underpinning strengths use as proposed by Govindji and Linley (2007), rather than their empirical results.

On the factorial level, the results showed that all items loaded significantly and sufficiently on their respective factors at both time points. All standardized factor loadings loaded significantly on their respective factors and ranged from 0.63 to 0.81 at Time 1 and 0.65 and 0.78 at Time 2. This exceeds the suggested cut-off criteria of 0.40, as Asparouhov and Muthén (2009) and Kline (2011) suggested. Further, no cross-loadings were present, item uniqueness was acceptable (>0.10 but <0.90; *p* < 0.01), and the average variance extracted was more than 50% for both factors at both time points (Asparouhov and Muthén, 2009; Kline, 2011). Further, all items showed a corrected item-total correlation coefficient larger than 0.3 (ranging from 0.56 to 0.77), implying that all items belong to their respective factors. This contrasts with other studies where a single factor of strengths use was reported. In the majority of international studies, several modifications to the SUS scale (such as correlating error terms, and item parceling) were required to enhance model fit and to increase measurement quality (c.f. Wood et al., 2011; Huber et al., 2017; Bu and Duan, 2020; Vuorinen et al., 2020). Enhancing model fit through statistical modification artificially inflates data-model fit but does not address the theoretical reasoning why the instrument did not perform as intended (McNeish et al., 2018). These modifications to the instrument also change the theoretical foundation on which the instrument is built, making comparisons to other studies improbable. Given that no modifications were made to artificially inflate model fit or measurement quality within the current sample, it would seem as though the two-factor model shows more promise.

Finally, the level of internal consistency at both the lower- and upper bound levels for all constructs at both time points suggest that the SUS was a reliable measure of strengths use. This is inline with other findings that showed high levels of internal consistency for the overall strengths use factor in the USA (Govindji and Linley, 2007; Wood et al., 2011), Germany (Huber et al., 2017), China (Bu and Duan, 2020), Finland (Vuorinen et al., 2020), South Africa (Mahomed and Rothmann, 2020), and the U.K. (McTiernan et al., 2020). The second-order factorial model could therefore be used as a reliable measure for Affinity for Strengths and Strengths Use Behaviors within the current context.

### Longitudinal Measurement Invariance and Factor Mean Comparisons

The results further showed that strict longitudinal measurement invariance of the SUS could be established over 3 months. Both the components (Affinity for Strengths and Strengths Use Behaviors) and overall strengths use factorial model was therefore measured (and interpreted) equally across time. This implies that the SUS showed similar factor structures, factor loadings, intercepts, and residual errors over time. Therefore, the data provide support for the stability of the SUS over time. When strengths use is assessed at two different time points, the mean difference indicates actual changes over time (Wong and Wong, 2020), rather than changes in the meaning of the constructs (Duncan et al., 2013). Meaningful comparisons between means and growth trajectories can, therefore, be made over time (Duncan et al., 2013).

No mean differences in neither strengths use, nor its components were reported within the current study. This shows that strengths use remained relatively stable over time (Duncan et al., 2013). This is in line with the assumption proposed by Peterson and Seligman (2004) and Govindji and Linley (2007) that strengths are considered psychological traits and that both the trait and its active use remain relatively stable over time. The stability in both the affinity for and active use of strengths would remain unchanged unless individuals are exposed to- or are engaging in strengths-based developmental initiatives (Seligman, 2012; Huber et al., 2017).

These findings are also relevant for long-term studies on strengths use like within intervention research. When employing longitudinal analytical strategies such as Latent Growth Modeling, where there are multiple measurement occasions, the input matrix of factors is large (Widaman et al., 2010). This leads to convergent problems and/or results in various statistical artifacts, which affects the interpretation of the results (Duncan et al., 2013; Wong and Wong, 2020). To reduce the complexity of these models, researchers would either parcel items or create mean scores to simplify the measurement models at the different time points within the study (Widaman et al., 2010). However, item parceling affects measurement invariance assessments at an item level, producing biased results (Meade and Kroustalis, 2006). Item parceling in longitudinal research should only be considered if there is a strong theoretical argument for such or when strict longitudinal measurement invariance has previously been established (Widaman et al., 2010; Duncan et al., 2013). Therefore, establishing strict longitudinal measurement invariance in the current study supports other researchers to parcel items on the scale when used in similar populations. However, these findings would need to be replicated in other populations to establish firmer conclusions.

### The Relationship Between Strengths Use and Study Engagement

The final objective of the paper was to establish criterion validity through relating Strengths Use to Study Engagement. First, concurrent validity was established by showing that Strengths Use at both Time 1 and Time 2 was positively related to engagement at the same time stamps. Further, predictive validity was established by showing that Strengths Use at Time 1 predicted Study Engagement at Time 2. The results imply that when a student can activate his/her strengths during their studies, it would lead to higher levels of study-related engagement. According to Van Woerkom et al. (2016b) this is because when individuals use their strengths, it aids them to live more authentically and therefore acts as an energizing mechanism. When students use their strengths during their studies, it leads to more inspiration, enthusiasm, excitement, and dedication to their study-related content (Seligman, 2012). Active strengths use therefore, has an invigorating effect (Huber et al., 2017). The results are aligned to several studies showing that higher levels of active strengths use lead to increased study and work-related engagement (Ouweneel et al., 2011; Seligman, 2012; Stander et al., 2015; Kwok and Fang, 2020). The SUS can therefore, be used as a measure to predict study engagement.

### Limitations and Recommendations

Although the study provides some unique insights, it is not without its limitations. First, the sample is relatively small and drawn from a single Dutch student population from a single Dutch University. This implies that the results may not be generalizable to other contexts or even institutions. It is suggested that the study be replicated in other educational contexts to further investigate the viability of the SUS as a measure of strengths use. Second, the interpretation of what is considered a strength was left to the participant. Therefore it is questionable if the SUS in deed measures “natural capacities coming from within that we yearn to use, that enable authentic expression, energize us and belong to positive traits and/or psychological capacities/talents refined with knowledge and skills” as articulated by Govindji and Linley (2007, p. 147). Although considered a strength of the instrument to measure strengths use in a general way, without providing a clear definition of what a strength is, could possibly lead to statistical artifacts within the data. This is because participants could understand strengths use as character strengths, talents, skills, abilities, or any other behavior pertaining to doing someone really well. It is suggested that the definition of a strength, as articulated by Govindji and Linley (2007), be included in the instructions to participants in the future. Further, it is suggested that a qualitative, open-ended question be added to the SUS requesting participants to both describe their definition of a strength, and provide 3 practical examples of their own strengths. This would aid in standardization in interpretation between participants.

Third, only student engagement was used as a metric to investigate criterion validity. Given that student engagement is a single (self-report) factor, future research should consider including “hard” or “objective” criterions such as academic performance or academic throughput. Fourth, the sample consisted out of predominantly males. Future studies should aim to include a more even distribution in terms of gender. Fifth, future research should investigate the convergent and discriminant validity of the SUS. Evidence for convergent validity could be tested by comparing the SUS with a measure of personal resilience, the 10-item Connor–Davidson Resilience Scale (CD-RISC) (Campbell-Sills and Stein, 2007). Evidence for discriminant validity could be tested by differentiating the SUS from a measure of personal emotional intelligence (e.g., the 16-item Wong and Law Emotional Intelligence Scale; Wong and Law, 2002). Additionally, future research should also investigate the correlation between the quantitative responses on the SUS with qualitative perceptions about the connotation they gave to strengths use when they responded to the items. This relates to what Alexandrova (2017) refers to as tracking the measurement of a construct as it is understood and endorsed by the respondent.

Sixth, it is suggested that more diverse population groups be considered for future validation studies. The SUS would benefit from a large scale cross-cultural validation study to determine if strengths use is seen and measured the same between cultures. Finally, future research should investigate the psychometric properties of a short-form SUS for rapid use by researchers and practitioners.

## Conclusion

Strengths use is a crucial factor to consider when designing both educational programmes and positive psychological interventions at universities. The current study shows support for the use of the SUS as a practical means to assess strengths use and to track the effectiveness of strengths use interventions within higher education environments.

## Data Availability Statement

The data employed in this study is available upon reasonable request from suitably qualified individuals and will be provided without undue reservations. Data management is aligned to the requirements of the General Data Protection Regulation.

## Ethics Statement

The authors declare that the research was conducted in the absence of any commercial or financial relationships that could be construed as a potential conflict of interest. Secondary data were employed in this study and the APA guidelines on Ethical Research practices were adhered to. All procedures performed in this study were in accordance with the ethical standards of the institutional requirements and in line with the Declaration of Helsinki. Written, informed consent was obtained from all participants before being permitted to complete the questionnaires. Participation in the study was entirely voluntary, participants were informed of their rights and responsibilities, and that they had the right to withdraw at any time. Given the nature of the study, no ethical clearance was required by the institution.

## Author Contributions

All authors listed have made a substantial, direct and intellectual contribution to the work, and approved it for publication.

## Conflict of Interest

The authors declare that the research was conducted in the absence of any commercial or financial relationships that could be construed as a potential conflict of interest.

## Footnotes

^1^Following the guidelines from the International Test Commission regarding the use and adaption of tests across cultures (Muñiz et al., 2013), before administration, the 14 items were piloted in a small group of master students to verify their clarity (*n* = 5). Based on feedback from the group, one item of the original instrument (STU_3 “I play to my strengths”) needed to be rephrased (“I pursue goals and activities that are aligned to my strengths”) in order to improve its comprehension within the Dutch context.

^2^The Strengths Use Scale was found to be comprised of two factors, Affinity for Strengths and Strengths Use Behaviors, and it was important and necessary for us to test a second-order factorial model to assess the hierarchical nature of the SUS. After first finding support for the first-order factorial model of the SUS items loading on the two first-order latent factors (Affinity for Strengths, Strengths Use Behaviors), we found support for a second-order factorial model in which the two first-order factors load onto the second-order factor, Overall Strengths Use. This therefore suggests that the SUS can be useful for measuring overall strengths use.

^3^For copyright purposes, several items were redacted. Items are, however, numbered and presented in the same order as in Govindji and Linley (2007) and Wood et al. (2011).
